# Outcome of transforaminal epidural steroid injection for lumbar radiculopathy: initial three-year experience at Upendra Devkota Memorial-National Institute of Neurological and Allied Sciences, Nepal

**DOI:** 10.1186/s41016-020-0184-5

**Published:** 2020-02-07

**Authors:** Pratyush Shrestha, Lojima Subba, Prity Agrawal, Subash Lohani

**Affiliations:** Upendra Devkota Memorial-National Institute of Neurological and Allied Sciences (UDM-NINAS), Bansbari, Kathmandu, Nepal

**Keywords:** Lumbar, Prolapsed intervertebral disc, Radiculopathy, Transforaminal epidural steroid injection

## Abstract

**Background:**

The prevalence of low back pain with radiculopathy in general population varies from 9.9% to 25%, which can be due to bony narrowing of the lateral recess or due to prolapsed intervertebral disc. Transforaminal epidural injection of a mixture of long-acting anaesthetic (bupivacaine) and particulate steroids (depomedrol) has been a treatment modality in patients not responding to initial physiotherapy and neuropathic pain medications.

**Methods:**

To analyze the effectiveness of transforaminal epidural steroid injection (TFESI) in the treatment of low back pain with radiculopathy, a retrospective case series evaluating the records of patients that received TFESI (1 mL 0.5% bupivacaine +1 ml/40 mg depomedrol) under C-arm guidance from January 2015 to December 2018 (3 years) at Upendra Devkota Memorial-National Institute of Neurological and Allied Sciences (UDM-NINAS), their lumbo-sacral MRI and the pre-procedure, 1-week and 3-month numeric pain rating scale, were analyzed. Successful treatment (reduction of pain scale by more than 50% of baseline at 3 months) in the patients with bony recess stenosis and those with prolapsed intervertebral disc was compared.

**Results:**

Out of 67 patients that received TFESI, 35 (52.23%) could be followed up. The mean age was 55.8 ± 14.39 years and 51.3% were females. 68.57% had L5 and 20% had S1 radiculopathy. Bony recess stenosis was seen in the aged 40% and PIVD was the cause of radiculopathy in 60%. The median duration of radicular pain prior to intervention was 3 months. TFESI was effective as the mean numeric pain scale before injection was 8.97 ± 1.32 which reduced to 3.91 ± 3.23 (paired *t* test *p* value < 0.001) at 1 week post injection and 3.23 ± 3.34 (paired *t* test *p* value < 0.001) at 3 months post injection. Twenty-six of the 35 patients (75.29%) had more than 50% pain relief compared to baseline at 3 months and were satisfied. Nine patients continued to have pain; however, only one required a surgical intervention. The effectiveness of TFESI was not significantly different in different ages (Fisher’s exact test *p* value 0.182) and in different anatomic levels (Fisher’s exact test *p* value 0.241). Six out of eight patients with bony recess stenosis benefited as compared to 14 out of 19 patients with PIVD, though it was not statistically significant (Fischer’s exact test *p* value 0.688). There were no adverse events recorded.

**Conclusion:**

TFESI is a safe and efficacious treatment modality in patients with radicular low back pain especially in aged patients in whom surgery under general anaesthesia is not free from risk.

## Background

Low back pain is one of the most common reasons for absence from work and physical limitation worldwide and affects 80% of the general population at some point in their lifetime [1, 2]. Lumbar radiculopathy (sciatica) with a prevalence of 9.9% to 25% is less prevalent than low back pain alone and is characterized by back pain radiating down the knees to the foot and toes, with variable neurological findings [3]. We lack systematic Nepalese data in this regard, but radiculopathy was present in 48.5% of patients with low back pain in patients undergoing MR imaging in a tertiary care centre in Kathmandu [4]. Compared to low back pain alone, sciatica is associated with more pain and disability, use of health resources and poorer quality of life [5]. According to the European guidelines [6, 7], the initial management of radicular low back pain is similar to nonspecific back pain; however, in this subgroup of patients, further treatment with spinal injections and surgeries is better defined compared to nonspecific back pain [5].

Radicular pain occurs not just due to mechanical compression but due to the release of neurochemical and inflammatory mediators at the target site [1]. Epidural injection can be done through inter-laminar, caudal, or transforaminal approach, whereby, local anaesthetics, steroids or a combination of the two can be delivered at the inflamed site. The transforaminal route is the best target-specific route to deliver the treatment agents to the ventral epidural space and dorsal root ganglion [8, 9]. Anti-inflammatory effect, neural membrane stabilization effect and modulation of the peripheral nociceptive effect are the probable ways by which the medications act around the inflamed nerve [10]. Various systematic reviews and randomized trials have proven the efficacy of TFESI for chronic radicular back pain [8, 11]. As various neurosurgical, orthopaedic and pain units around the country, at UDM-NINAS, TFESI had been instituted on patients with chronic radicular pain not responding to the initial management of modification of activities, different exercises, analgesics and neuropathic medications, physical therapy and manual manipulations for the past 3 years. This review was to analyze the outcome of our intervention, an appraisal of the technique routinely practiced as well as to identify complications so as to improve on the way we managed chronic lumbar radiculopathy in the days to come.

## Methods

This was a retrospective case series of patients that received TFESI for chronic radicular backache from January 2015 to December 2018 in UDM-NINAS, a tertiary care neuro centre in Kathmandu, Nepal. Patients that did not respond to standard initial management, those not willing for surgical intervention and aged patients with risk for microscopic discectomy under general anaesthesia were given the option of targeted lumbar root block. The procedure was carried out by a neurosurgeon or a neurosurgical trainee under direct supervision of a neurosurgeon in the radiology suite of the hospital

### Procedural steps for transforaminal epidural steroid injection

**Figure 1:**
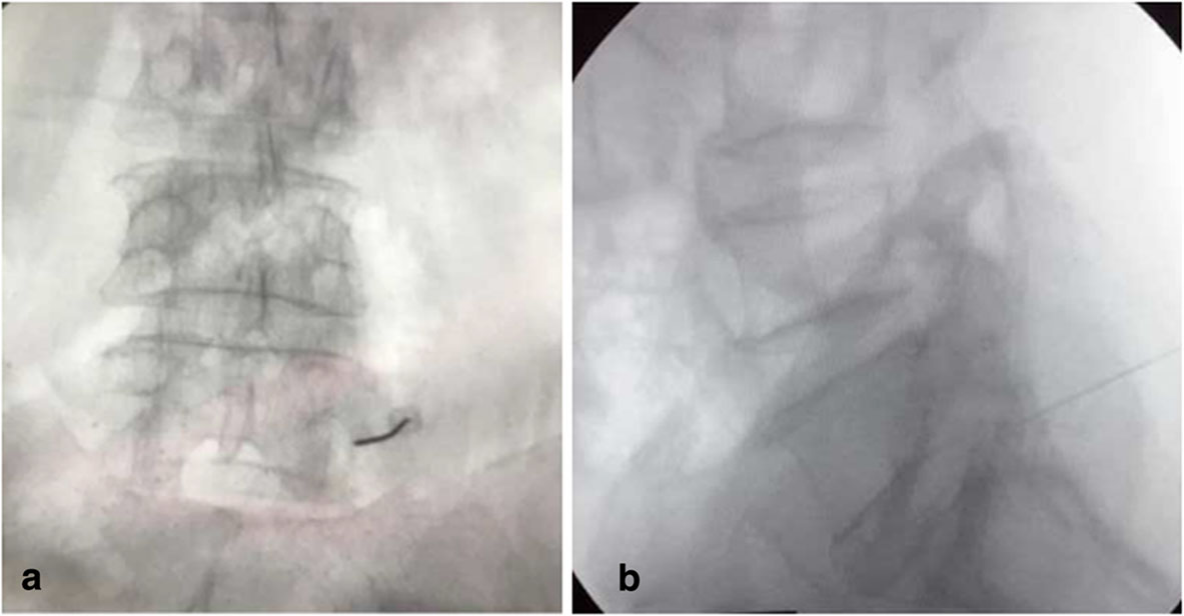
AP (**a**) and lateral (**b**) views of the left L5 root block

The intervention was done on day-care basis after adequate counselling, consenting and ruling out underlying coagulopathy. Patients were made to lie prone on the radiolucent table after checking for allergy to the iodine dye. Antero-posterior (AP) subpedicular approach was used to deliver the treating medicine in the traditional safety triangle, which is the epidural space just caudad to the inferior margin of the pedicle, immediately superior, lateral and anterior to the targeted exiting nerve [12, 13]. Local anaesthetic was infiltrated 3 to 5 cm lateral to the midline on the affected side after localizing the level on fluoroscopic lateral view. Then on AP view, the segmental level was optimized by squaring off the superior endplate, which required a cranial tilt of the C-arm for L4–5 and L5–S1 levels. A twenty-two-gauge spinal needle was then introduced through the anaesthetized skin to the target point which is the posterior surface of the vertebral body near the midline aspect of the inferior border of the pedicle (6 o’clock position or slightly lateral) above the targeted nerve [1]. Once the bone was contacted, a AP fluoroscopic shot was taken to ascertain that the needle tip was not medial to the midline of the pedicle; the position of the needle tip was confirmed with a lateral fluoroscopic shot to be posterior to the vertebral body just below the pedicle (Fig. 1)

To target the S1 root, the upper end plate of the sacrum was squared off with a cranial tilt of the C-arm and the caudal border of the anterior half of the S1 pedicle passing the needle through the posterior S1 sacral foramen. A lateral fluoroscopic shot is taken to confirm the needle tip close to the floor of the sacral canal but never ventral to it [1].

If the needle tip inadvertently came in contact with the exiting nerve, the patient felt a sharp twitch going down his leg (for which the patient was earlier made aware of), and the tip was slightly withdrawn but the procedure continued as this was a confirmatory sign of vicinity to the exiting nerve.

One millilitre of iodine dye was then introduced after re-aspiration to rule out vascular injection and peri-neuro-seathogram analyzed to ascertain perineural flow of dye along with spread of dye in the ventral epidural space. Premixed 1 mL of 0.5% bupivacaine and 40 mg (1 mL) of depomedrol was then gradually injected after re-aspiration to rule out vascular injection again.

Immediate effect of the treatment medicine was assured by making the patient walk in the radiology suite. The patient was discharged after observing him/her for 4–6 h post procedure with counselling regarding modification of activities, different exercise, physical therapy and follow-up 1 week post procedure.

### Data collection and analysis

Information of all patients who received TFESI from January 2015 to December 2018 was retrieved from the operation theatre record book and further information of imaging findings and contact number from the hospital electronic record system. A performa for the telephonic inquiry had been made and agreed by the authors. An independent assessor, here author LS, contacted the subjects by phone and performed standardized interview to determine the outcomes. Numeric pain rating scale (NPRS) from 0 to 10 was used to characterize radicular pain before TFESI, at the end of the first week and 3 months following the procedure. The duration of pain relief, the need for another TFESI and the need for surgical intervention at any point in time or any inadvertent complications as limb weakness infection were enquired. Improvement in the numeric pain score by more than 50% of the baseline at 3 months post TFESI was regarded as a successful intervention.

The records of MRI images were reviewed by a consultant radiologist, here author PA, where the cause of the radiculopathy was differentiated mostly by prolapsed intervertebral disc or by the bony narrowing of the lateral recess. The outcome of the intervention in these two subgroups at 3 months compared. Chi-squared test (Fischer’s exact test) was done to evaluate the outcome in these two categories, and a *p* value of < 0.05 was taken as statistically significant.

A master table was made in the Statistical Package for the Social Sciences (Window version 20.0; SPSS Inc, Chicago, IL, USA) which was used for analyzing frequency distribution and all statistical analysis (Additional file 1). Paired *T* test was used to compare the pre-procedure, 1-week and 3-month NPRS, and a *p* value of < 0.05 was taken as statistically significant

## Results

A total of 67 patients received TFESI over the study duration of 3 years. As per the routine practice, immediate post procedure pain relief was confirmed in all patients, and there were no patients that did not have immediate relief of radicular low backache. Only 35 (52.23%) of these patients could be contacted for telephonic interview by the independent assessor; hence, analysis as a whole was confined on these 35 patients.

The mean age was 55.8 ± 14.39 years, with the youngest being 32 years and the oldest being 80 years. Aged patients in whom surgical intervention could not be offered following failure of conservative treatment due to risks of general anaesthesia were given the option of TFESI; 10 (28.57%) of the 35 patients were 70 years and above. There was no sex predilection in the treated population with 51.3% being females.

Regarding the level of involvement, 68.57% of patients had involvement of the L5 root due to pathology at the L4–L5 level and 20% had S1 radiculopathy due to pathology at L5–S1 level. In two patients, the L4 root was involved and in two (5.71%), both L5 and S1 were involved. Only these two patients received TFESI at two levels, the rest at a single level. Nearly half (17 of 35) of the patients had the left side affected, the rest 18 had a right-sided involvement.

The median duration of symptoms of radicular low backache after which the patient received TFESI was 3 months with interquartile range 11 months. TFESI was not offered to patients with radicular symptoms less than a month. The maximum duration of radicular pain after which TFESI was done was 8 years.

To assess the effectiveness of the TFESI, a numeric pain rating scale from 0 to 10 was used. The numeric pain rating scale for the radicular pain was recorded before the intervention. The mean numeric pain rating scales before the intervention, at the end of 1 week and after 3 months along with the standard deviations were as follows (Table 1).

**Table 1:** Numeric pain rating scale before, at the end of a week and 3 months post TFESI

Numeric pain rating scale			Paired *t* test (*p* value)
	Mean	Standard deviation	
Before root block	8.9714	1.31699	< 0.001
At the end of 1 week	3.9143	3.23920	
At the end of 3 months	3.2286	3.34388	

The mean numeric pain rating scale dropped markedly following the intervention at the end of 1 week. The pain scale continued to remain low at the end of 3 months following the procedure.

A successful intervention has been defined as the reduction of radicular pain by more than 50% of baseline at 3 months following TFESI. Twenty-six of the 35 patients (75.29%) had more than 50% pain relief compared to baseline at 3 months and were satisfied. Nine patients continued to have pain; however, only one required a surgical intervention. Twelve out of 14 patients with bony recess stenosis benefited as compared to 14 out of 21 patients with PIVD, though it was not statistically significant (Fischer’s exact test *p* value 0.194). There were no adverse events recorded.

In 9 (25%) patients out of 35, TFESI was not effective. In all of these 9 patients, the numeric pain rating scale was not reduced adequately at 1 week post injection, though all patients responded to TFESI immediately. Only one of these patients required surgery and the rest continued other forms of conservative management.

There were no major complications in this limited series as epidural hematoma, infection or arterial injection as sterility was paid attention to and aspiration before injecting the medication was done routinely. Some patients, however, felt dizzy when made to walk after the injection which improved on its own after they rested supine for some time. It was a routing to keep the patients in the hospital for 4 h for observation after the injection.

### Outcome difference in the young and old

Aged patients with sciatica cannot be treated with microscopic discectomy when the initial conservative measures fail due to the risks of general anaesthesia. We evaluated the efficacy of TFESI in this subgroup (Table 2).

**Table 2:** Outcome at 3 months in young and aged population

Age	Improvement by > 50% at 3 months		Total	Fisher’s exact test
	No	Yes		
< 70 years	8	17	25	*p* value = 0.182
≥ 70 years	1	9	10	
Total	9	26	35	

Nine (90%) of 10 patients that were ≥ 70 years responded well with TFESI compared to only 68% in the younger subgroup as they had a reduction in numeric pain rating scale by more than 50% at 3 months. This however was not statistically significant.

### Outcome difference in L5 and S1 root blocked

On a technical note, the trajectory to block the L5 root differs from that to block the S1 route as has been discussed in the methodology section. To analyze if this difference affected the outcome, the outcome of patients who underwent singular L5 or S1 root block were analyzed. Four patients (two where L4 root were blocked and two where both L5 and S1 were blocked) were excluded from the analysis (Table 3).

**Table 3:** Analysis of the outcome at 3 months based on the root blocked

Root blocked	Improvement by > 50% at 3 months		Total	Fisher’s exact test
	No	Yes		
L5	5	19	24	*p* value = 0.241
S1	3	4	7	
Total	8	23	31	

Only four (57.14%) of the seven patients with S1 problem benefited at 3 months compared to 19 (79.17%) of the 24 patients; this, however, was not statistically significant.

### Outcome difference in the radiculopathy due to PIVD or bony recess stenosis

We tried to evaluate the effectiveness of TFESI in the two subgroups where the radiculopathy was due to bony recess narrowing or due to prolapsed intervertebral disc. For this, an independent Consultant radiologist reviewed all MR imaging of the lumbo-sacral spine and decided which of the two the cause of radiculopathy in the patients was. There were patients who had bony recess narrowing as well as some PIVD. It was under the jurisdiction of the radiologist to decide which the more important cause in these scenarios was. Of the 35 patients who could be interviewed, we could retrieve 27 (77.14%) MRI lumbo-sacral spine records.

The mean age of patients in whom PIVD was the cause of radiculopathy was 53.37 ± 13.28 years (19 patients) compared to 61.5 ± 15.1 years (eight patients) in whom bony narrowing of the recess was the cause. Our presumption that older patients tended to have more of bony narrowing of lateral recess was however proven wrong as *p* value (independent sample *t* test) was 0.175, though the mean age of patient with bony narrowing of the lateral recess was higher.

To evaluate the effectiveness of TFESI in these two subgroups, a chi-squared (Fisher’s exact) test was done. The following is the tabulation (Table 4).

**Table 4:** Outcome difference at 3 months in patients with radiculopathy with PIVD or bony recess narrowing

Cause of radiculopathy	Improvement in NPRS by more than 50% at 3 months		Total	Fisher’s exact test
	No	Yes		
PIVD	5	14	19	*p* value, 0.688
Bony recess narrowing	2	6	8	
Total	7	20	27	

Fourteen (73.68%) out of 19 patients who had a PIVD continued to have improvement at 3 months compared to 6 (75%) of patients with bony recess stenosis. The outcome was comparable in the two subgroups, and TFESI was not selectively better for one subgroup or the other.

## Discussion

Back pain is the fifth most common reason individuals seek medical care in USA, and annually, 30 to 50 billion dollars is spent on healthcare to treat it annually [14]. The subgroup of patients with radicular low back pain have more severe pain and disability, longer recovery period and absence from work, according to epidemiologic studies [15, 16]; however, clinical studies suggest a more favourable clinical course and natural history [17, 18]. Though limited, the treatment of radicular back pain is better defined, and with timely identification and institution of spinal injections and surgical intervention, healthcare costs and long-term disability can be markedly reduced in this subgroup [5].

TFESI is an effective form of minimally invasive treatment in patients with unilateral radicular pain due to herniated lumbar disc or spinal stenosis [19]. In the recent years, it has been extensively used in patients with radicular low backache [20]. The epidural space in the lumbar spine can be reached through interlaminar, transforaminal or caudal approaches [21, 22]. Schaufele and colleagues conducted a case-control study comparing interlaminar and transforaminal approaches and concluded that the latter resulted in a better short-term pain improvement and fewer long-term surgical interventions [22]. Ghai and colleagues [21] conducted a randomized, double-blind, active control trial comparing these two approaches and concluded that the parasagittal interlaminar approach was equally effective in achieving pain relief and functional improvement and that it had a better safety profile and technical ease.

In the transforaminal approach, there are two commonly practiced techniques, the anterior-posterior subpedicular approach and oblique Scotty dog subpedicular approach. Kaliya-Perumal and colleagues have highlighted in details the procedural steps of both the approaches [13]. To reduce radiation exposure to the radiation, we used the anterior-posterior subpedicular approach at our centre. In another review article, Mandel and colleagues discuss the anatomy of the lumbar neural foramen and key considerations in planning TFESI [23]. The posterior lateral approach and Kambin’s triangle approach were discussed. At our centre, we targeted the traditional safety triangle keeping the needle tip just inferior to the pedicle and superior, anterior and lateral to the exiting nerve [23]. Atluri and colleagues [24] have reviewed the literature and discussed cases of 10 cases of paralysis from TFESI due to injection in the radicular artery.

They have stressed the danger of superior and anterior position of the needle tip during TFESI. Keeping this dreaded complication in mind, we avoided the needle tip position medial to the middle of the pedicle and always aspirated before injecting the particulate steroids.

The agent used for this intervention, however, is highly debated. Ng and colleagues randomized patients indicated for TFESI to get methyl prednisolone and bupivacaine or bupivacaine alone. There was no difference in the outcome at 3 months [25]. A systematic review by Roberts and colleagues concluded that TFESI was better than placebo in treating radicular backache and that it could even avert surgical intervention. It was not useful, however, in patients with failed back syndrome and when there was fibrosis documented around the targeted nerve [8]. Manchikanti and colleagues compared lidocaine and saline with lidocaine and betamethasone and found the outcome with steroids was not superior than a local anaesthetic alone at 2 years [11].

Leung and colleagues [26] have published their experience of TFESI in 232 patients. In their series, 14 patients (6%) had multiple level involvements; it was 5.7% in our series. The benefit lasted for 1 to < 3 weeks in 15%, 3 to 12 weeks in 15.9% and > 12 weeks in 39.7% of the patients. Our result deferred in this regard, as in 75.29% of our patients that received TFESI, there was > 50% pain reduction at the end of 3 months, and these patients continued to be physically active. The rest nine patients did not have adequate response even at the end of the first week.

Kennedy and colleagues [27] followed up patients who received TFESI beyond 5 years prospectively, found that even though the success rate of TFESI at 6 months was high, most of the patients had recurrence of symptoms in the subsequent 5 years. The follow-up of our study ranges from 3 months to 3 years; hence, we need to continue the follow-up to understand the outcome of TFESI for a longer duration. In this study too, only 50% of the subjects were reachable for telephonic follow-up. We could follow up 35 (52.23%) out of 67 patients that received TFESI, which highlights the drawback of a retrospective study.

Adiley and colleagues [28] compared the effect of single TFESI for L4–5 and L5S1 paramedian disc herniation and found that the TFESI was more effective for L4–5 paramedian disc herniation. This was comparable to our series where TFESI for the S1 root (L5S1 PIVD) was successful only in 4 (57.14%) out of 7 patients compared to 79.17% for the L5 root (L4–5 PIVD). This is probably due to the more difficult trajectory to reach close to the exiting S1 root and also probably due to the anatomical difference in these levels.

TFESI can be effective in treating radicular backache so much so as to avert the need of a surgical intervention. The premise is that once the acute pain is taken care of, the patient continues physical therapy, activity modification to strengthen the paraspinal muscles and hence prevent further recurrences. The result of the NERVES trial by Wilby and colleagues [29] is highly awaited as this is the first trial to evaluate the effectiveness of microdiscectomy to local anaesthetics and steroid administered via TFESI. This will help to develop an evidence-based treatment strategy for patients with sciatica and hopefully check the rampant practice of microscopic discectomy as seen in recent years.

Our study has a few limitations. It is a depiction of the early outcome following TFESI and we need to further follow up our patients for a longer duration to establish the efficacy. Manchikanti and colleagues have highlighted the short-term efficacy of this technique; however, the evidence of the long-term efficacy is only moderate [30]. In our study, we could follow up only 35 (52.23%) of the 67 patients intervened. As ours is a tertiary centre where we get patients from very remote areas of the country, follow-up is a major problem. As TFESI was done as an outpatient basis, the records are not as strong as that for inpatients. This is the early result of a single centre and in the future multi-centre studies could establish better results.

## Conclusion

TFESI is a safe and efficacious treatment modality in patients with radicular low back pain especially in aged patients in whom surgery under general anaesthesia is not free from risk. The relief from the activity limiting radicular pain gives the patient opportunity for further exercising and strengthening the low back muscles so as to avert the need of surgical intervention.

## Supplementary information

**Additional file 1.** Master Table.

## Data Availability

The SPSS master worksheet has been submitted to the journal as additional submission.

## Abbreviations

AP: Anterior-posterior
MRI: Magnetic resonance imaging
NERVES trial: Nerve Root Block vs Surgery Trial
NPRS: Numeric pain rating scale
PIVD: Prolapsed intervertebral disc
TFESI: Transforaminal epidural steroid injection
UDM-NINAS: Upendra Devkota Memorial National Institute of Neurological and Allied Sciences
